# Motorcyclists' reactions to safety helmet law: a qualitative study

**DOI:** 10.1186/1471-2458-9-393

**Published:** 2009-10-20

**Authors:** Fereshteh Zamani-Alavijeh, Shamsaddin Niknami, Eesa Mohammadi, Ali Montazeri, Fazlollah Ghofranipour, Fazlollah Ahmadi, Shahrzad Hejazi Bazargan

**Affiliations:** 1 Department of Health Education, Tarbiat Modares University, Tehran, Iran; 2 Department of Nursing, Tarbiat Modares University, Tehran, Iran; 3 Department of Mental Health, Iranian Institute for Health Sciences Research, ACECR, Tehran, Iran; 4 Department of Psychiatry and Biobehavioral Sciences, Charles Drew University of Medicine and Science, Los Angeles, California, USA; 5 University of California, Los Angeles (UCLA), Los Angeles, California, USA

## Abstract

**Background:**

Extensive body of the literature reveals that proper use of helmets is an effective way to reduce the severity of injuries and fatalities among motorcyclists. However, many motorcyclists do not use safety helmet properly. This study aimed to empirically explore reactions of motorcyclists to the safety helmet laws, in Iran.

**Methods:**

Qualitative data were collected via four focus groups and 11 in-depth interviews. Participants were 28 male motorcyclists who never used a safety helmet during rides, and 4 male police officers. All transcripts, codes and categories were read for several times to exhaust identifiable major themes. During this process data were reduced from text to codes and themes.

**Results:**

Five major themes emerged from the data analyses, including themes related to the following: (1) circumventing or dodging police officers; (2) simulating a helmet wearing behavior; (3) accepting the probability of receiving a ticket; (4) taking advantage of the police neglect and carelessness; and (5) using a cheap or convenient helmet.

**Conclusion:**

Our findings suggest certain levels of reckless driving among the participating motorcyclists in this study. They also point to a system of law enforcement that operates haphazardly and fails to consistently penalize those who deviate from it. Further studies are needed to investigate how "risks" are perceived and relate to "reactions", and how a 'culture of masculinity' may encourage risk tolerance and a disposition toward lawlessness and carelessness among male motorcyclists. Also, there is a need for the development and implementation of multidimensional interventions that would offer socio-culturally sensitive educational and motivational messages to the motorcyclists and the in-service traffic-enforcement officers in Iran.

## Background

Traffic injuries in many countries are considered "the neglected epidemic" [1]. Over half of all road traffic collisions involve motorcyclists; the risk of incurring severe injuries or fatalities are ten times higher among this group than users of four-wheeled vehicles [2,3]. A report from the World Health Organization (WHO) identifies motorcyclists among the most vulnerable groups for road traffic injuries [4]. Motorcyclists have also been identified at risk for head trauma [5-7].

In Iran, the motorcycle is an important means of transportation and its use is highly common among men [8]. In 2005, motorcycles made up about 40% of the country's registered vehicles. In the capital city, Tehran, the number of monocycles has increased to more than 2 million during the last few years [2]. A report from WHO shows that almost 70% of motorcycle deaths in Iran are due to head injuries resulting from the non-use or improper use of helmets [2]. It is also documented by Naghavi [9] and Roudsare [10] that motorcycle-related head injuries are most prevalent among men.

Many studies have shown that the proper use of safety helmets is an effective way to reduce the severity of injuries and fatalities among motorcyclists during collisions [7,8,11-13]. In most countries, including Iran, the safety helmet laws require motorcyclists to use a helmet at all times while riding a motorcycle [14]. Although this law was enacted in Iran many years ago, it was seriously enforced not until 2002 [15]. Currently, motorcyclists who are guilty of disobeying the helmet law are subject to monetary fines and in some cases to imprisonment. If these offenders accumulate convictions, their motorcycles are seized and their licenses are suspended for a period of time. Concurrently, public media such as radio and television inform motorcyclists about the safety features of the helmets and also recommend their use [2].

Several social, political and economic factors might have had a contributory role in the enforcement of the helmet law in Iran, including the following:

(1) With the end of Iraq-Iran war at 1994, Iran experienced an upward trend in the number of registered companies manufacturing motorcycles, i.e. from two companies in 1994 to thirty in 2000 (a period called the "reconstruction period"). This trend led to a dramatic increase in the number of motorcycles in the country [16].

(2) Due to the uncontrolled production of cheap and unsafe motorcycles by private industries, as well as the availability of cheap gas due to government subsidies, motorcycles became an affordable means for making money, especially, among the low-income and unemployed population. Therefore, there was an increase in the use of motorcycles for the transportation of passengers and goods in the cities and rural areas [17].

(3) National data suggested a low rate of helmet use among motorcyclists. A national survey of motorcyclists in several cities in Iran indicated that of the 92% of the motorcyclists who owned a helmet, only 13% were using it. [2]. Another study conducted in Tehran concluded that only 8.6% of injured motorcyclists were wearing a helmet at the time of the crash. Also, helmet use varied by the time of the day and the season of the accident; use was more frequent (25.2%) between 16:00 and 19:00 hours, and more frequent in winter (34.8%) and autumn (26.0%) than in spring (16.0%) and summer (23.2%) [8].

(4). Government officials and researchers reported an upward trend in the number of registered vehicles, road traffic injuries, and road traffic deaths between 1995 to 2002, with an enormous contribution by motorcyclists in fatal, and even more significantly, in non-fatal injuries [15,18].

(5) The last factor which led to the enforcement of the safety helmet law, had to do with the enormous economic burden of traffic-related deaths and collisions, costing the nation approximately $6 billion a year [2].

Relative to the extent of the efforts that have been put to enforce the safety helmet law in Iran and most countries, there is a paucity of research that explores the reactions of motorcyclists to this law. Such research could be an important first step toward the development of an effective intervention for the prevention or reduction of injuries among this population [3,19].

The specific aim of this qualitative study was to empirically explore reactions of motorcyclists to safety helmet laws. Identification of theses reactions are a first step toward revealing the underlying factors related to non-compliance with the use of safety helmets among this segment of the population.

## Methods

### Participants and data collection

Data were collected using individual in-depth interviews and focus-group discussions [20,21]. Recruitment of participants for the focus groups and the in-depth interviews were based on convenient and purposeful sampling. Decisions regarding the number of in-depth interviews were determined by data saturation. Individuals were eligible to participate in the study if they were male (nearly all motorcyclists in Iran are male and the Police Academy is an all-male academy), used a motorcycle as the main means of transportation, and reported either "not using" or "rarely using" a safety helmet. Eligibility criteria for participating in either the focus group or the in-depth interview were the same. In total, four focus groups with twenty-one motorcyclists, and eleven in-depth interviews with seven motorcyclists and four police officers (n = 32) were held between January 2007 and February 2008. Approval for the study was obtained from the Tarbiat Modares University. Written informed consent was obtained from all subjects prior to their participation in the study.

The focus-group discussions had an open and semi-structured format, each lasting approximately 45 to 70 minutes, and began with a few general questions to initiate conversation among the participants in regard to their reactions to the mandatory safety helmet law (i.e. What do you think about the safety helmet law?" "Do you comply with it?"; "If not, how do you handle police officers when you get caught?"). Focus group facilitators used probes as necessary to stir up discussions on the subject matter. Therefore, while methods of initiating focus-group discussions were all the same across all groups (i.e. using the same general question to initiate discussion), different probes were used depending on responses given by the participants. Participants were encouraged to talk freely and to not hesitate in sharing their experiences, feelings, and behaviors with the group. As mentioned above, to capture various reactions to the mandatory helmet law, we also conducted eleven in-depth, semi-structured interviews (i.e. seven with motorcyclists and four with police officers who were recruited specifically for this purpose), each lasting between 30 to 60 minutes.

### Analysis

Focus-group discussions and in-depth interviews were tape-recorded, transcribed verbatim, and analyzed subsequently. The comments of participant were kept in their original form and were translated verbatim, for the purpose of this paper. Subsequently, they were reviewed and revised by a bilingual health expert for readability.

Data analysis involved the repeated reading of transcripts in combination with listening to the recorded interviews. Thematic analyses were used, in which a theme was defined as "an abstract entity that brings meaning and identity to a current experience and its variant manifestations" [22]. First, transcripts were read and reread in order to get an overall impression. Second, investigators identified meaning-units from each transcript and performed initial coding according to the participants' phrases. Initial codes were identified by at least two co-investigators, and codes were then categorized according to their similarities and differences [22]. Transcripts, codes and categories were read several times by various members of the research group to exhaust identifiable major themes. During this process, data were reduced from text to codes and themes.

In addition, a random selection of over 40% of the transcripts were read and coded separately by two study advisors who held regular meetings to discuss and resolve coding issues. The study team also met on a regular basis to discuss plausible data interpretations and to ensure that the qualitative data analysis was systematic and verifiable, as recommended by the experts [23-25]. The evaluation criteria for establishing the trustworthiness of qualitative data (i.e. credibility, dependability, confirmability, and transferability) were also considered [23]. This process was iterative, with new data used to assess the integrity of the developing analysis. There was constant comparison within and across categories and across interviews where each code or category was checked against the rest of the data to establish and refine categories that reflected the nuances of the data [26].

## Results

Of the 32 participants in the study, 28 were male motorcyclists who reported "not using" or "rarely using" any safety helmet while riding a motorcycle, and four were members of an all-male police academy who reported patrolling areas popular to the motorcyclists. Five major themes emerged from the data analyses, including themes related to the following: (1) *circumventing or dodging police officers*; (2) *simulating helmet wearing behavior*; (3) *accepting the probability of receiving a ticket*; (4) *taking advantage of police neglect and carelessness*; and (5) *using a cheap or convenient helmet*.

The "***circumventing or dodging police officers" ***theme was more frequent among respondents who were not as concerned with the harmful consequences of riding without a helmet as they were with how to avoid confrontations with the police officers. These respondents seemed to know how to escape police officers by avoiding certain routes and hours of the day during which officers were more likely to be present. A motorcyclist said, "*I know which streets are patrolled by the officers; therefore I avoid them*." These riders acknowledged that sometimes motorcyclists warn each other of the locations where police officers are stationed so that other riders would avoid them. They explained that under those circumstances the best resort or escape, which often is risky, is to make a quick u-turn, or drive on the sidewalks, or drive in the opposite direction of an oncoming traffic. A rider who had 17 years of motorcycling experience said, "*I can easily escape the police. They chase me for a while, but then, they give up, because I drive through the alleys or in the opposite side of the streets*." Study participants who were the members of police force admitted that occasionally they have to cease chasing un-helmeted motorcyclists to protect the safety of the *pillion *passengers or people on the streets. They argued that motorcyclists often take advantage of these situations. One of the policeman in our study remarked, "*When other motorcyclists see that we are citing a rider without a safety helmet, they speed up and get away. Sometimes, they flee riding in the opposite side of a one-way street or on a sidewalk or through a narrow alley. Although we chase them, we often have to give up because we are afraid that they will crash into a car, or overrun an innocent passer-by*." Another police officer said, "*If we pursue motorcyclists by chasing them, we might put their lives and the lives of others in danger. Also, some motorcyclists don't have a license plate on their vehicle or any vehicle registration documents which makes it even more difficult for us to legally pursue their cases."*

Respondents who owned a safety helmet (standard or non-standard) but were in the habit of not wearing it unless they feared the presence of a police officer supported the ***"simulating helmet wearing behaviors" ***theme. These respondents said they would carry a helmet while riding their motorcycles and use it only when the situation deems it necessary (i.e. to avoid getting a ticket). One of the participants remarked, "*I wear the helmet because I am scared of the police. I don't wear it if I don't see any police officers around. I usually buckle its straps around my shoulder; as soon as I see a police officer I quickly wear it pretending that I was wearing it all along. I do not even buckle the straps unless a police sees me*." Another participant said, *"My helmet is always with me, it is just that I don't wear it, unless I have to*." In support of this theme one of the police participants said the following: *"We have been instructed to cite all the un-helmeted motorcyclists. However, when they see us approaching them, immediately they remove their helmet from the handle of the motorcycle or their shoulder and wear it. This method of use is no good because, first, they do this while riding which is risky and can cause a crash; second, they don't use the helmet correctly as directed by the manufacturer and leave the straps unbuckled, and third, people should want to wear the helmet on their own, for their own safety, not because they are afraid of us. The helmet law cannot fully protect them from the road traffic injuries if they are not motivated to comply it."*

As was mentioned in the above theme, some participants reacted to the safety helmet law by not using it unless they had to. But, there were other participants who fit into the "***accepting the probability of receiving a ticket" ***theme. These were motorcyclists who opposed using safety helmets altogether. These riders were willing to confront the consequences of their behaviors by paying the fines associated with their violations. They claimed that police officers are rarely stationed on the routes on which they commute daily. Therefore, they usually don't see any motorcyclists getting stopped or cited for not wearing a safety helmet. They also believed that even if they get caught by a police officer, they are willing to pay the monetary fine because it is not much. One respondent said, "*I really like riding without a helmet and I won't enjoy it as much if I have to wear one. I am prepared to pay the fine. I really cannot stand wearing a helmet. So far, I have been caught a few times and each time I have paid the monetary fine. To me it was worth it." *Police participants in the study, also, argued that for the well-to-do individuals the mandated fine for not using a safety helmet is too low to be a motivation for the use of safety helmets. They claimed that the helmet law doesn't fully prevent this group from suffering road injuries because the individuals in this group enjoy riding with no helmet. One of the police officers said, *"Usually when I stop motorcyclists from the uptown, they are ready to pay the fine. Once one of them once told me, come on; give me the citation and let me get on with my fun."*

There were also a number of respondents who endorsed the "***taking advantage of police neglect and carelessness" ***theme. These participants believed that not all members of the police force consistently enforce mandated traffic laws. They argued that police officers don't regularly fine and/or seize the violator's motorcycle. According to one participant, "*There is always the possibility of not getting a ticket even if one gets caught for not wearing a helmet*." These respondents claimed that haphazard enforcement of the helmet law by police officers sometimes encourage them not to adhere to the law. A few motorcyclists argued that they can persuade police officers not to give them tickets. They would do so by convincing officers that either they or their passenger were sick and needed urgent care. They believed that some police officers are easy to convince. A 42 year-old motorcyclist explained, "*I never use a safety helmet and when I get caught and the officer wants to fine me I tell him that I have a respiratory problem and show him the ID card which would verify it. Most officers would then overlook my offence and let me go*." Some motorcyclists stated that police officers have a tendency to overlook their offences when a female is a pillion passenger. A motorcyclist said, "*When my wife is on pillion of my motorcycle I never use a helmet and officers don't stop me*." Police officers seem to agree that sometimes they do overlook non-compliant motorcyclists. They admitted that some riders can convince them that they are from a low-income family and cannot afford paying the fine. One officer expressed that it is hard for him to fine a motorcyclist who begs for forgiveness and cries for leniency. Another motorcyclist remarked, *"It is hard to fine or seize someone's motorcycle who claims he has no money and that the motorcycle is his sole property and source of earning money. They beg for forgiveness and convince you that if you fine them their wife and children will suffer from hunger."*

There were a number of participants who indicated that non-standard helmets were more convenient to use. According to their descriptions a convenient helmet had the following features: (a) was cheaper, (b) was lighter in weight, and therefore, easier to carry around, (c) was thinner, (d) provided partial facial coverage thus offering better ventilation, and (e) was commonly accessible in the market. These respondents supported the ***"using a cheap or convenient helmet" ***theme. They were not against the helmet laws *per se *but were concerned about the uncomfortable structure and expensive market price of the standard safety helmets. According to these participants since laws requiring helmet use were passed, their non-standard prototypes (sometimes 20-times cheaper) is commonly available in the markets. They argued that they evaluate and compare the advantages and disadvantages of standard and non-standard helmets before purchasing one. One respondent said, "*Non-standard ones are cheaper and more comfortable, have better ventilation so I don't feel hot when wearing them, but of course, they don't provide much protection. On the other hand, the standard ones give us full protection, but they are much more expensive, have less ventilation, and difficult to breathe in, altogether much more uncomfortable*." Another motorcyclists expressed, "*The standard helmet is much safer but I don't have one because it is too expensive, makes me feel hot and, and it is difficult to breathe in. Of course I have a helmet, but one that is cheaper and has no cover in the front. It is not standard, but at least I don't get a ticket anymore. Recently these helmets are more available in the market and are more popular among motorcyclists. They are good enough to trick the officers*." One respondent claimed, "*Police officers don't even care or look at the type or the way we wear the helmet, they just try to enforce its use and give us ticket if we don't wear it."*

## Discussion

This study is one among a few qualitative studies that attempt to explore the reactions of motorcyclists to the safety helmet law in Tehran, Iran. Results from the in-depth interviews and focus-group discussions helped to delineate the following themes: (1) *circumventing or dodging police officers*; (2) *simulating helmet wearing behaviors*; (3) *accepting the probability of receiving a ticket; *(4) *taking advantage of police neglect and carelessness*; and (5) *using a cheap or convenient helmet*. These results reveal risky reactions to the mandatory helmet law among the participating motorcyclists. Indeed, research on safety helmet laws shows that such laws do not necessarily lead to lower traffic road injuries [12,27].

Findings of this study suggest that the reported reactions to the helmet law could be the consequences of a system that operates haphazardly to enforce this law. In other words, the system has failed to consistently penalize those who deviate from this law. Motorcyclists in this study revealed that even when a violator of the helmet law is caught it does not necessarily mean that he will be cited for the violation. A study in northeast Thailand revealed that proper enforcement of the helmet law increased its compliance among motorcyclists, although wearing a helmet did not significantly reduced fatalities among the injured motorcyclists [28]. A study from Indonesia showed that motorcyclists did not wear their helmet when police officers were not present [29]. In two mid-sized cities in China, 75% of the riders reported they would wear a helmet not to prevent head injuries but to "cope with the police officers" [3]. The same study also reported higher rates of helmet use by the motorcyclists during the hours of the weekdays when police officers were more likely to be present [3].

Other system-level factors which provoked participants' reactions to the helmet law were "cost", "inconvenience", and the "uncomfortable fit of the helmet." As shown in this study, some motorcyclists use non-standard safety helmets and/or use it improperly to compensate for its high cost and inconvenient fit. Previous studies report that the non-standard helmet and an improper use of safety helmet provides limited protection during a collision [28,30]. Despite this fact, the use of non-standard helmets is common among motorcyclists in various countries [17,28,31]. For example, in provincial areas of China, 34% of the riders did not use a helmet at all, and among those who did wear a helmet, 33.7% did not buckle its straps properly [3]. Following an introduction of the mandatory helmet law in California, about 10% of motorcyclists used a non-standard helmet, and some purchased these less protective helmets unknowingly [30]. Other studies have cited lack of ventilation, heat, and the uncomfortable structure of standard helmets as reasons for non-use [32,33].

The availability and accessibility of cheap and non-standard helmets in the market, as reported by the participants in this study, calls for the government to play a more regulatory role. Discontinuing the manufacture and distribution of non-standard helmets, subsidizing the cost of standard helmets for low-income families, and enforcing regulations that ban the import of non-standard helmets, may increase the likelihood of purchasing and using standard safety helmets among motorcyclists.

It might be suggested that the reactions reported in the study were the result of risk appraisals associated with the consequences of helmet non-use or misuse. However, we did not directly assess participants' risk perceptions, therefore, we cannot categorize the participants into any specific risk groups. Wilde [34] argues that individuals subconsciously and/or consciously weigh the advantages and disadvantages of choosing target levels of risks. He suggests that the cognitive process of risk assessment is determined by the individual's past experiences, assessment of his/her potential risks for getting into an accident, and the degree of confidence the individual has in his/her skills to handle a vehicle. According to the Wilde's theory, people look for a certain level of thrill in their lives; therefore, behaviors with zero risk usually don't catch their attention. To change the risky reactions of riders to the helmet law, it is important to identify where along the continuum of risk-taking they are and how ready they are to change those reactions. This may allow interventionists to properly match risk-reduction programs with the readiness level of the target audience. Respondents in our study seem to fall somewhere along this continuum of risk assessment. Further studies are needed to assess the role of "risk perception" in provoking various reactions to the helmet laws.

Furthermore, studies are needed to assess the impact of the "culture of masculinity" in promoting risky reactions toward safety helmet laws, among male motorcyclists. This "culture of masculinity" promotes uptake of certain values and behaviors among men. For example, men are expected to be emotionally distant and rational, and behaviorally, aggressive, tough, and autonomous [35]. Men who adopt these beliefs and behaviors are more likely to engage in activities that compromise their health and threaten their longevity [36]. From the social constructionist perspective, social practices in which men engage are the means for demonstrating masculinity [37]. Within this perspective, motorcycling or joining the police force in Iran facilitates the presentation of a masculine persona among men.

The use of motorcycles in the construction of masculinity underlines the importance of gender-specific interventions. This kind of intervention can build on what the culture of masculinity idealizes -- that men are expected to provide for their family as the main breadwinner -- and so empower men as responsible individuals.

Some investigators suggest motorcyclists should be motivated to properly and consistently use safety helmets [28]. Others suggest educational programs are needed to inform consumers of the specifications of standard and non-standard safety helmets, as well as, their proper usage and injury prevention functions [3]. Another group contends that reinforcement measures provide deterrent to non-compliant motorcyclists when they are enforced appropriately and consistently [38]. Nonetheless, the endorsement of the safety helmet law without providing adequate socio-culturally sensitive educations and interventions may result in unfavorable outcomes. As shown by the results of this study, these laws might lead to further reckless behaviors.

Reinforcement measures along with educational programs that consider the overall culture of motorcycle riding in Iran are highly recommended [8]. These programs can promote and motivate safe riding by increasing motorcyclists' awareness of the danger of risky and reckless ridings, informing them about existing laws and the risks of being detected, explaining the physical, legal, and financial consequences of non-complaint reactions to the helmet law. Intervention programs should strive to overcome possible barriers related to: (1) A 'culture of masculinity' which might promote risk tolerance among male motorcyclists and a disposition toward lawlessness and carelessness; and (2) motorcyclists' misinterpretation of the laws. In Iran, especially after the Islamic revolution of 1979, religion and family have played an important role in holding individuals responsible for their own health and safety of the others. In the Quran one is taught; "to stay away from self-harm" and "caution not to harm others" [39,40]. However, there is a paucity of scientific evidence showing an association between the role of family and religion in promoting helmet safety use among motorcyclists. A retrospective study among 166 junior and high school students reported that students with a "high family norm of bicycle helmet use" were more likely to use helmets than student who came from families with a "low family norm". Similarly, student who grew up in a bicycle-friendly community were more likely to use helmet more often [41]. A different study reported that a parent-child intervention yields positive outcome in the helmet use of their adolescent child [42]. Motivational interventions are needed to evaluate the impact of the local culture, family role, and religious messages in promoting the use of safety helmets among motorcyclists.

At the same time, a lack of consistency in enforcing the traffic laws by the enforcement officers (according to our findings) calls for the system-level interventions. Regular law-enforcement training programs and/or workshops for the in-service enforcement officers can help law enforcement officers to better understand and share problems and concerns in providing good law-enforcement services. Training goals should be based on periodic assessments of enforcement activities of the officers so that the content of training matches with the officers' existing needs. Findings of this study imply that traffic enforcement officers could advantage from training workshops that will increase their knowledge and skills to act decisively and motivate them to improve their compliance with the traffic-law enforcement goals and established policies.

This study has the inherent limitation of any qualitative research -- the findings are limited by design and not generalizable to the larger motorcyclist population. Other limitations of the study included the following: (1) we did not directly assess the variable 'perception of risk', which might influence certain reactions among the participants; and (2) this study could not offer any gender-related information about the reactions of motorcyclists to the safety helmet law.

Despite these limitations, our method has followed four essential aspects of a qualitative analysis [43]: (1) the inclusion criteria were relevant to the research question; (2) data collection methods (i.e. focus-group discussions and in-depth interviews) were appropriate for the specific aim of the study; (3) data collection processes were rigorous and comprehensive to support saturation and robust descriptions of the information collected; (4) data was analyzed appropriately and results were corroborated by using multiple reviewers to ensure that participants' viewpoints were adequately interpreted.

Furthermore, our results offer some insights into research that is not well established, and therefore, can be built upon in further studies. Risk appraisals may be performed on un-helmeted riders to better understand the perception of the physical, legal, and financial factors that contribute to the risky reactions to the helmet laws.

## Conclusion

Our findings shed some light on various non-compliant reactions of the motorcyclists to the safety helmet law and suggest certain levels of reckless driving among them. They also point to a system of law enforcement that operates haphazardly and fails to consistently penalize those who deviate from it. This is an important step in assessing the underlying factors that provoke motorcyclists to either not use or misuse a safety helmet. Further studies are needed to investigate how "risks" are perceived and relate to "reactions", and how a 'culture of masculinity' may encourage risk tolerance and a disposition toward lawlessness and carelessness among male motorcyclists.

The present study also alludes to the need for the development and implementation of comprehensive and multidimensional interventions that would offer socio-culturally sensitive educational and motivational messages to the motorcyclists and the in-service traffic-enforcement officers in Iran. Further studies should evaluate the short and long term effectiveness of these programs in improving prevention and the reduction of road traffic injuries among motorcyclists.

Results from this study also offer valuable suggestions for developing culturally relevant assessment tools to quantitatively measure reactions and factors related to non-compliance reactions among this segment of the population. A culturally tailored tool can assess knowledge, attitudes and behaviors of motorcyclists in respect to the mandatory helmet law, as well as frequency, time, and the locations where safety helmets are more or less likely to be utilized.

## Competing interests

The authors declare that they have no competing interests.

## Authors' contributions

FZ was the main investigator and contributed to development of research protocol, implementation of the research, and drafted the manuscript. SN supervised the study and scientific integrity of data collection and revision of the manuscript. EM was advisor to the study, contributed to the interpretation of data and revising the manuscript. AM was advisor to the study, contributed to the interpretation of the data and revising the manuscript. FG contributed to the interpretation of data and revising the manuscript. FA participated in the design of the study and interpretation of data. SB was involved in critical review, intellectual content, and revisions of the manuscript. All authors have read and approved the final manuscript.

## Pre-publication history

The pre-publication history for this paper can be accessed here:


